# Kushenol C Prevents Tert-Butyl Hydroperoxide and Acetaminophen-Induced Liver Injury

**DOI:** 10.3390/molecules26061635

**Published:** 2021-03-15

**Authors:** Byoung Ok Cho, Jang Hoon Kim, Denis Nchang Che, Hyun Ju Kang, Jae Young Shin, Suping Hao, Ji Hyeon Park, Feng Wang, Yun Ji Lee, Seon Il Jang

**Affiliations:** 1 Research Institute, Ato Q&A Co., LTD, Jeonju-si 55069, Korea; dkgk0608@naver.com (H.J.K.); sjy8976@naver.com (J.Y.S.); 2 Institute of Health Science, Jeonju University, Jeonju-si 55069, Korea; chedenis88@gmail.com; 3 Department of Herbal Crop Research, National Institute of Horticultural & Herbal Science, RDA, Eumsung 27709, Korea; oasis5325@gmail.com (J.H.K.); Yoong0625@korea.kr (Y.J.L.); 4 Department of Health Management, Jeonju University, Jeonju-si 55069, Korea; hsuping0211@163.com (S.H.); wlgusliza@naver.com (J.H.P.); w-971@163.com (F.W.)

**Keywords:** kushenol C, Nrf2, Akt, antioxidant, OGG1, liver injury

## Abstract

*Sophora flavescens*, also known as Kushen, has traditionally been used as a herbal medicine. In the present study we evaluated the ameliorative effects of kushenol C (KC) from *S. flavescens* against tBHP (tert-Butyl hydroperoxide)-induced oxidative stress in hepatocellular carcinoma (HEPG2) cells and acetaminophen (APAP)-induced hepatotoxicity in mice. KC pretreatment protected the HEPG2 cells against oxidative stress by reducing cell death, apoptosis and reactive oxygen species (ROS) generation. KC pretreatment also upregulated pro-caspase 3 and GSH (glutathione) as well as expression of 8-Oxoguanine DNA Glycosylase (OGG1) in the HEPG2 cells. The mechanism of action was partly related by KC’s activation of Akt (Protein kinase B (PKB)) and Nrf2 (Nuclear factor (erythroid-derived 2)-like 2) in the HepG2 cells. In in vivo investigations, coadministration of mice with KC and APAP significantly attenuated APAP-induced hepatotoxicity and liver damage, as the serum enzymatic activity of aspartate aminotransferase and alanine aminotransferase, as well as liver lipid peroxidation and cleaved caspase 3 expression, were reduced in APAP-treated mice. Coadministration with KC also up-regulated antioxidant enzyme expression and prevented the production of proinflammatory mediators in APAP-treated mice. Taken together, these results showed that KC treatment has potential as a therapeutic agent against liver injury through the suppression of oxidative stress.

## 1. Introduction

The dried roots of *Sophora flavescens*, also known as Kushen, and one of the oldest medicinal herbs used in traditional Chinese medicine, are used for the treatment of a variety of ailments including toxicity removal, parasite elimination and diuresis induction. *Sophora flavescens* is also used in the traditional Chinese medicine for the treatment of gastrointestinal hemorrhage, skin diseases and pyretic stranguria, and has been confirmed in many studies to have antitumor, antiviral and anti-inflammatory properties [1,2,3,4]. Phytochemical studies have also confirmed the presence of quinolizidine alkaloids and prenylated flavonoids, which, interestingly, have demonstrated pharmacological activities as antitumor, antiviral and anti-inflammatory agents, thus confirming the pharmacological benefits of Kushen as depicted in traditional Chinese medicine [5,6,7]. One of the prenylated flavonoids isolated from Kushen is Kushenol C (KC). However, limited data exist on the biological activities of KC. One study reported that KC possesses greater antioxidant activities, by preventing the generation of reactive oxygen species in a liver cell line, compared to other prenylated flavonoids such as kushenol A, 8-prenylkaempferol, formononetin and 8-prenylnaringenin isolated from the roots of *S. flavescens* [8]. We previously reported that KC inhibited the activation of signal transducer and activator of transcription 1 (STAT1), STAT6, and nuclear factor kappa B (NF-κB) in a stimulated macrophage cell line and upregulated the activation of nuclear factor erythroid 2-related factor 2 (Nrf2) and Akt in the phosphoinositide 3-kinase (PI3K)-Akt signaling pathway in a skin cell line, thereby conferring anti-inflammatory and antioxidative stress properties to KC [7]. KC was shown to inhibit the activities of cytochrome P450, the drug-metabolizing enzyme and drug transporter that plays a central role in the metabolism and elimination of drugs in the liver [9]. In the present study, we investigated whether KC confers protection to the liver against tert-Butyl hydroperoxide (t-BHP)-induced and acetaminophen (APAP)-induced liver oxidative injury and sought to understand its mechanism of action.

## 2. Materials and Methods

### 2.1. Plant Material

The roots of *S. flavescens* were purchased from Jeongeup herbal medicine shop, Korea, on April 2015. The species was identified by J.H. Kim. A voucher specimen (NIHHS-1) was deposited in the herbarium of the Department of Horticultural and Crop Environment, National Institute of Horticultural and Herbal Science (NIHHS).

### 2.2. Extraction and Isolation

**Figure 1:**
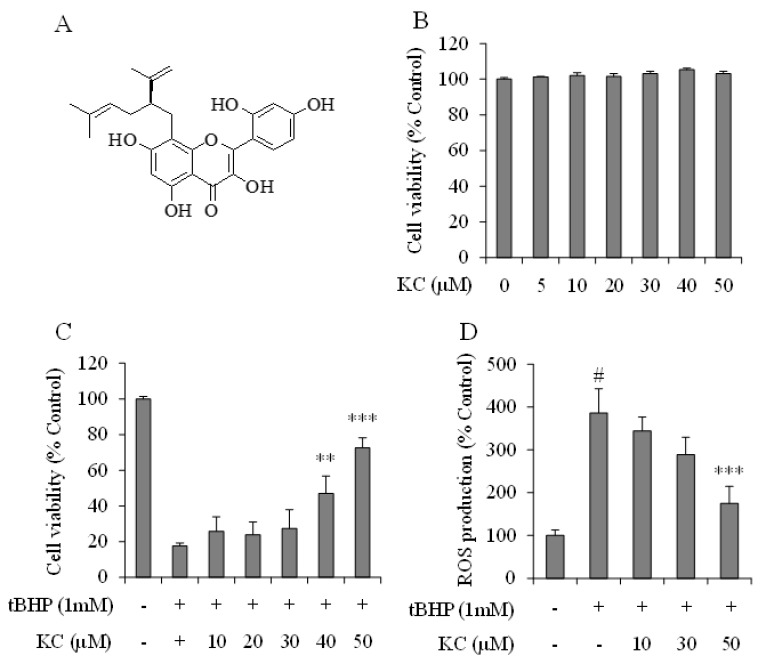
Effect of kushenol C on cell viability and reactive oxygen species (ROS) production in tBHP-treated HEPG2 cells. (**A**) The Chemical structure of Kushenol C (KC). (**B**) Cells were treated with different concentration of KC and cell viability was determined using EZ-Cytox reagent. (**C**) Cells were preincubated with KC at different concentrations for 1 h and then treated with tBHP for 24 h. Cell viability was determined using EZ-Cytox reagent. (**D**) Cells were preincubated with KC at different concentrations for 1 h and then treated with tBHP for 1 h and ROS production was determined as described in the Materials and Method section. Error bars represent the mean ± SD, # *p* < 0.001 vs. Control, ** *p* < 0.01, *** *p* < 0.001 vs. tBHP-only treated cells.

The roots of *S. flavescens* were extracted three times with 72 L of 95% methanol (MeOH) at 27 °C for seven days. The concentrated methanol extract (770 g), suspended in distilled water (1 L), was fractionated by chloroform (CHCl3), ethyl acetate (EA) and water. 100 g of the EA fraction was chromatographed with silica gel column chromatography using a CHCl3-MeOH gradient system (from 1:0 to 1:4) to yield ten fractions (EA1-EA10). The EA7 fraction was separated by C-18 column chromatography using a water-MeOH gradient system (from 1:1 to 1:7) to achieve compound 1 (9 mg, KC, Figure 1A).

### 2.3. Chemicals and Reagents

EZ-Cytox reagent and KC (≥98%, HPLC) came from Dogenbio (Seoul, Korea) and ChemFaces (Wuhan, China), respectively. APAP, silymarin, tert-Butyl hydroperoxide (tBHP), ImmunoHistoMountTM, LY294002 PI3K/Akt inhibitor, ML385 Nrf2 inhibitor, and O8 OGG1 inhibitor came from Sigma-Aldrich (St. Louis, MO, USA). 6-carboxy-2′,7′-dichlorodihydrofluorescein diacetate (carboxy-H2DCFDA, C400) came from Invitrogen (Carlsbad, CA, USA). Antibodies for 8-Oxoguanine DNA Glycosylase (OGG1), Nrf2, Bcl-2, Bax, phosphor-p38, phosphor-JNK, and β-actin came from Santa Cruz Biotechnology (Santa Cruz, CA, USA). Antibodies for caspase-3, cleaved caspase-3, Akt, phosphor-Akt, and phosphor-ERK came from Cell Signaling Technology Inc. (Beverly, MA, USA). JC-1 dye and MEBCYTO apoptosis kits came from Thermo Scientific (Rockford, IL, USA) and MBL International (Nagoya, Japan), respectively. Horse serum, ImmPRESSTM HRP, and 3-amino-9-ethylcarbazole (AEC) peroxidase substrate came from Vector laboratory (Burlingame, CA, USA). All other chemicals used were of reagent grade and came from Sigma Chemical Co. (St. Louis, MO, USA) unless otherwise stated.

### 2.4. Cell Culture

HEPG2 cells were cultured in Dulbecco’s modified eagle medium (DMEM) supplemented with 10% fetal bovine serum (FBS) and 1% penicillin/streptomycin antibiotics at 37 °C under 5% CO_2_ in an incubator. The cells were subcultured at 80% confluence, and during the culturing period cells were counted and used in experiments.

### 2.5. Cell Viability Studies

Cell viability was performed using EZ-Cytox reagent. The HEPG2 cells (3 × 10^5^ cells/mL) were seeded in 96-wells plates for 16 h and treated with or without KC at various concentrations for 1 h, and further incubated with or without tBHP (1 mM) for 24 h. Then 10 μM of EZ-Cytox reagent was added to the wells and incubated for 4 h. The absorbance of each well was then measured using a spectrophotometer (Tecan, Männedorf, Switzerland) at 450 nm. The absorbance of each well corresponded with the HEPG2 cells’ viability and was calculated as the percentage of the control.

### 2.6. Measurement of Intracellular ROS

The HEPG2 cells were incubated KC at indicated concentrations for 1 h and then treated with tBHP (1 mM) for 1 h. The cells were incubated with 10 μM carboxy-H2DCFDA for 0.5 h and the cells were then washed in PBS and harvested. Intracellular ROS was immediately examined using a flow cytometer (Cytomics FC500; Beckman, Miami, FL, USA).

### 2.7. Western Blot Analysis

The HEPG2 cells were preincubated with or without 50 µM of KC for 1 h and then treated with or without 0.5 mM of tBHP for 12 h. Then cellular proteins were extracted in radioimmunoprecipitation assay buffer (RIPA buffer). The protein concentration was measured using Bradford’s assay and 30 µg of protein from each sample was subjected to Western blot analysis. For the Western blot analysis, the protein was separated on an SDS-PAGE gel by electrophoresis and electrophoretically transferred to a polyvinylidene fluoride (PVDF) membrane. The membranes were incubated with various primary antibodies overnight and after washing in tris-buffered saline with 1% Tween 20 (TBST) buffer, the membranes were further incubated with appropriate corresponding horseradish peroxidase-conjugated secondary antibodies. After further washing, the proteins on the membranes were visualized using an enhanced chemiluminescence reagent (Dogenbio). The membranes were stripped with Western blot stripping buffer (Thermo scientific) and incubated with actin antibodies and the immunoblotting procedure repeated. The protein bands were quantified using ImageJ analysis software and normalized to the expression of the internal control β-actin; the results were further normalized to the control.

### 2.8. Measurement of Cellular Glutathione

The HEPG2 cells were preincubated with or without 50 µM of KC for 1 h and then treated with or without 0.5 mM of tBHP for 12 h. The cells were harvested in cold buffer containing 50 mM MES, pH 6.5. and 1 mM EDTA. After centrifugation, at 10,000× *g* for 15 min at 4 °C the supernatant was removed and after deproteinization, glutathione (GSH) was measured using Cayman Chemical GSH assay kits (Ann Arbor, MI, USA) in accordance with the manufacturer’s instructions.

### 2.9. Flow Cytometry Analysis

The HEPG2 cells were preincubated with or without 50 µM of KC for 1 h and then treated with or without 0.5 mM of tBHP for 12 h. An apoptosis assay was performed using the MEBCYTO Apoptosis Kit according to the manufacturer’s instructions. In brief, the cells were trypsinized in PBS and resuspended in binding buffer. Annexin V- fluorescein isothiocyanate (FITC) and Propidium Iodide were added, mixed and incubated at room temperature for 15 min in the dark. After the incubation, binding buffer was added and the cell samples were measured using a flow cytometer (Cytomics FC500; Beckman, Miami, FL, USA).

### 2.10. Immunofluorescence Analysis

The HEPG2 cells were preincubated with or without 50 µM of KC for 1 h and then treated with or without 0.5 mM of tBHP for 12 h. Immunofluorescence staining was performed according to the kits manufacturer’s protocol (Thermo Scientific). In brief, after culture and treatment of cell of the cells, they were incubated at 37 °C for 15 min with the prepared staining solution. The cells were trypsinized and centrifuged at 400× *g* and the supernatant discarded. The cells were resuspended in assay buffer and washed once. After washing, the cells were suspended in assay buffer and 5 µL was transferred onto a glass slide for analysis by fluorescent microscopy.

### 2.11. Animals and Treatments

Specific pathogen-free male Balb/c mice (six weeks old) weighing 18–20 g were obtained from Orient Bio Inc. (Gwangju, Korea). They were housed in a room with standard environmental conditions of temperature 22 ± 2 °C, humidity of 50–60% and a 12/12 h light-dark cycle. The mice were fed with a commercial standard laboratory diet and water ad libitum. The experimental procedures were performed in accordance with the Jeonju University Institutional Animal Care and Used Committee guidelines (Approved No. JJU-IACUC-2018-2). The mice were randomly assigned into six groups with five mice per group as follows: group 1, normal control; group 2, APAP 500 mg/kg; group 3, APAP plus KC 1 mg/kg; group 4, APAP plus KC 10 mg/kg; group 5, APAP plus KC 20 mg/kg; group 6 (a positive control), APAP plus silymarin 50 mg/kg. KC and silymarin were prepared in saline. APAP was prepared in vehicle (1% Et-OH and saline). Groups 1 and 2 were administered the saline, and groups 3–6 were administered KC and silymarin orally every day for seven days. Three hours after the final administration, group 1 was treated intraperitoneally with the vehicle. Groups 2–6 were treated intraperitoneally with APAP at a dose of 500 mg/kg of body weight and fasted for 16 h. All groups were subsequently euthanized. Blood was obtained by cardiac puncture after the mice had been anesthetized with ether. The blood samples were allowed to solidify at room temperature for 30 min and then centrifuged to separate the serum at 2000 g for 15 min at 4 °C. Liver samples were also harvested and rinsed with ice cold saline. The liver samples were quickly frozen with liquid nitrogen and stored at minus −80 °C until used for further studies. A portion of each liver tissue was fixed in neutral buffered formalin for histopathologic examination.

### 2.12. Analysis of Serum AST and ALT

Activities of the hepatic enzymes aspirate aminotransferase (AST) and alanine aminotransferase (ALT) were determined using AST and ALT colorimetric assay kits (BioVision, Milpitas, CA, USA) following the manufacturer’s instructions.

### 2.13. Histopathologic Examination

Histopathological examination was performed as previously described by Cho et al., [10]. Hematoxylin and eosin (H&E) stain was used for evaluation of liver toxicity. Immunohistochemistry was performed using a cleaved caspase-3 antibody. Histopathological changes were analyzed under a light microscope (Leica, Wetzlar, Germany).

### 2.14. Analysis of Lipid Peroxidation and GSH Amount

Lipid peroxidation and GSH amount in liver tissues were determined using an MDA (malondialdehyde) ELISA (enzyme-linked immunosorbent assay) kit (Cell Biolabs, San Diego, CA, USA) and a GSH assay kit (United States Biological, Salem, MA, USA) in accordance with the manufacturer’s instructions, respectively.

### 2.15. Analysis of Liver SOD (Superoxide Dismutase) and Catalase

Liver SOD and catalase activities were determined using SOD and catalase assay kits (Cayman Chemical, Ann Arbor, MI, USA) in accordance with the manufacturer’s instructions.

### 2.16. Analysis of Serum TNF-α and IL-6

The concentrations of TNF-α (tumor necrosis factor- α) and IL-6 (interleukin-6) in serum were determined using an ELISA kit (R&D Systems, Minneapolis, MN, USA) in accordance with the manufacturer’s instructions.

## 3. Results

### 3.1. KC Showed no Cytotoxicity to HEPG2 Cells and Prevented tBHP-Induced ROS Production and Cytotoxicity on HEPG2 Cells

The effects of KC (chemical formula shown in Figure 1A) on cell viability of HEPG2 cells were first studied. The results presented in Figure 1B demonstrated that treatment of the cells with KC up to 50 µM alone did not significantly affect the viability of the cells. Then the effects of KC on tBHP-induced ROS production and cell death were investigated. The results of the effects of KC on tBHP-induced cell death are shown in Figure 1C. The results revealed that treatment of the cells with tBHP significantly decreased the viability of the cells, while pretreatment with KC at 10, 20, 30, 40, and 50 µM prevented the decrease in cell viability and significant preventions were obtained when the cells were treated with 40 and 50 µM KC. As presented in Figure 1D, when the HEPG2 cells were treated with tBHP alone, ROS production in the cells was significantly increased. However, when the cells were pretreated with KC at 10, 30, and 50 µM, ROS production in the cells was dose-dependently decreased and a significant decrease was obtained when the cells was treated with 50 µM of KC.

### 3.2. KC Prevented tBHP-Induced Apoptosis and Mitochondrial-Related Apoptosis Signals in HEPG2 Cells

**Figure 2:**
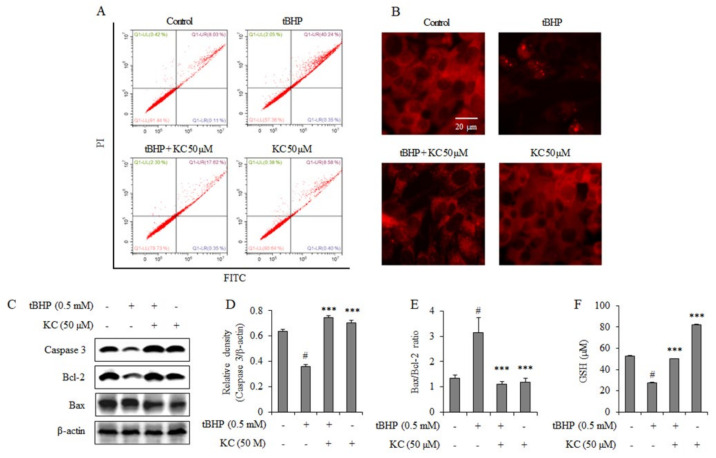
Effect of kushenol C on apoptosis, mitochondria membrane potential, caspase-3 and Bax/Bcl-2 expressions in tBHP-treated HEPG2 cells. Cells were preincubated with or without KC for 1 h and then treated with or without 0.5 mM of tBHP for 12 h. (**A**) Apoptosis assay was performed using an MEBCYTO (registered trademark of MBL International Corporation (Woburn, Ma, USA)) Apoptosis Kit. (**B**) Immunofluorescence staining was performed using a JC-1 mitochondrial membrane potential kit. (**C**) Caspase-3, Bcl-2, and Bax protein expression levels were investigated by Western blot assay and the band densities were analyzed using ImageJ analysis software, with respect to actin (**D**,**E**). (**F**) Intracellular glutathione (GSH) was measured using a Cayman assay GSH assay kit. Error bars represent the mean ± SD, # *p* < 0.001 vs. Control, *** *p* < 0.001 vs. tBHP-only treated cells.

Next, the effects of KC on tBHP-induced apoptosis and alteration in mitochondrial-related apoptosis signal were investigated. As shown in Figure 2A, flow cytometer analysis demonstrated that treatment of the cells with tBHP increased the apoptosis of HEPG2 cells, while preincubation with KC at 50 µM before treatment with tBHP prevented apoptosis in the cells. Treatment of the cells with KC at 50 µM alone did not have any effects on apoptosis of the cells when compared to the control. Immunofluorescence data presented in Figure 2B revealed that the cells treated with tBHP had significant loss of mitochondrial membrane potential, while with 50 µM of KC preincubation the loss in mitochondrial membrane potential was decreased in the tBHP-treated cells. Figure 2C,D, through Western blot analysis, showed that cells treated with tBHP had decreased expression of caspase-3, while preincubation of the cells with 50 µM of KC inhibited the decrease of caspase-3. Treatment of the cells with KC at 50 µM alone did not have any effects on caspase expression in the cells when compared to the control. Figure 2C also revealed that cells treated with tBHP showed degradation of antiapoptotic Bcl-2 and slightly increased Bax, while the cells that were preincubated with KC at 50 µM before treatment with tBHP showed no significant degradation of Bcl-2, while Bax was significantly degraded. Treatment with KC at 50 µM alone did not have any significant effects on Bcl-2, while Bax was also significantly decreased when compared to the control. The ratio of Bax/Bcl-2 expression significantly decreased in the cells with KC (Figure 2E).

### 3.3. KC Rescued tBHP-Induced Cellular GSH Depletion

The effects of KC in tBHP-induced GSH depletion in HEPG2 cells were investigated. As shown in Figure 2D, there was a depletion of GSH in the cells treated with tBHP, while the depletion of the GSH was significantly reduced when the cells were preincubated with 50 µM of KC before treatment with tBHP. Treatment of the cells with 50 µM of KC alone significantly increased GSH when compared to the control.

### 3.4. KC Prevented tBHP-Supression of the DNA Repair Protein OGG1

**Figure 3:**
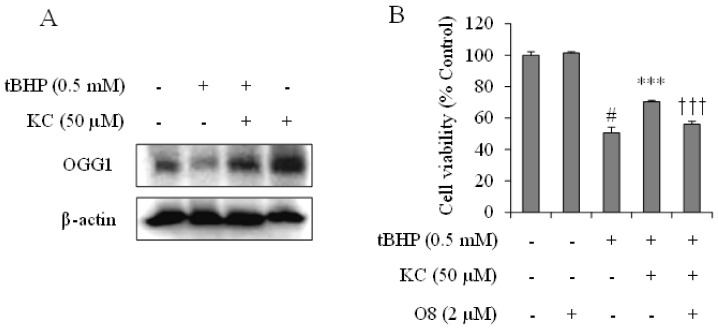
Effect of kushenol C on OGG1 expressions in tBHP-treated HEPG2 cells. (**A**) Cells were preincubated with or without KC for 1 h and then treated with or without 0.5 mM of tBHP for 12 h. OGG1 expressions protein levels were investigated by Western blot assay. (**B**) Cells were preincubated with KC at different concentrations and O8 (the OGG1 inhibitor) for 1 h and then treated with tBHP for 12 h. Cell viability was determined using EZ-Cytox reagent. Error bars represent the mean ± SD, # *p* < 0.001 vs. Control, *** *p* < 0.001 vs. tBHP-only treated cells, ††† *p* < 0.001 vs. tBHP plus KC treated cells.

The effects of KC on OGG1 expression in tBHP-treated HEPG2 cells were investigated. As demonstrated in Figure 3, OGG1 expression in the cells was decreased when the cells were treated with tBHP. However, preincubation of the cells with KC at 50 µM before treatment with tBHP prevented the suppression of OGG1 expression. In addition, treatment of the cells with KC alone upregulated the expression of OGG1 when compared to the control cells. The results in Figure 3B further revealed that while preincubation of the tBHP-treated cells with KC alone prevented cell death, preincubation of the cells with KC and O8, the OGG1 inhibitor at noncytotoxic concentration, did not prevent the tBHP-induced cell death.

### 3.5. KC Upregulated the Activation of AKT and Nrf2 Expression in tBHP-Treated Cells

**Figure 4:**
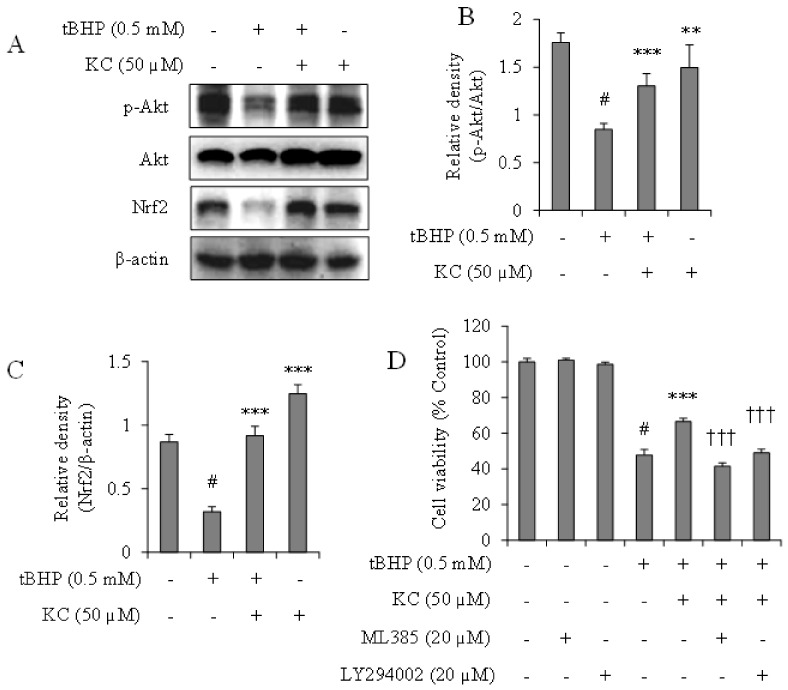
Effect of kushenol C on AKT and Nrf2 expressions in tBHP-treated HEPG2 cells. (**A**) Cells were preincubated with or without KC for 1 h and then treated with or without 0.5 mM of tBHP for 12 h. AKT and Nrf2 protein expression levels were investigated by Western blot assay and the band densities were analyzed using ImageJ analysis software, with respect to actin (**B**,**C**). (**D**) Cells were preincubated with KC at different concentrations and/or AKT and Nrf2 inhibitors for 1 h and then treated with tBHP for 12 h. Cell viability was determined using EZ-Cytox reagent. Error bars represent the mean ± SD, # *p* < 0.001 vs. Control, ** *p* < 0.01, *** *p* < 0.001 vs. tBHP-only treated cells, ††† *p* < 0.001 vs. tBHP plus KC treated cells.

The effects of KC on the activation of AKT and expression of Nrf2 in tBHP-treated cells were investigated. As reported in Figure 4A–C, there was a decrease in the expression of activated AKT (p-AKT) and Nrf2 expression in HEPG2 cells treated with tBHP alone. When the cells were preincubated with 50 µM of KC before treatment with tBHP, significant increases in the expressions of p-AKT and Nrf2 were observed. In addition, treatment with KC alone maintained the expression levels of p-AKT and Nrf2 in the cells when compared to the control cells. Further investigations with potent inhibitors of AKT (LY294002) and Nrf2 (ML385) revealed that while KC prevented tBHP-induced cell death, copreincubation of the tBHP-treated cells with KC and ML385 or LY294002 at noncytotoxic concentration did not prevent tBHP-induced cell death (Figure 4D).

### 3.6. KC Upregulated the Activation of MAPK in tBHP-Treated Cells

**Figure 5:**
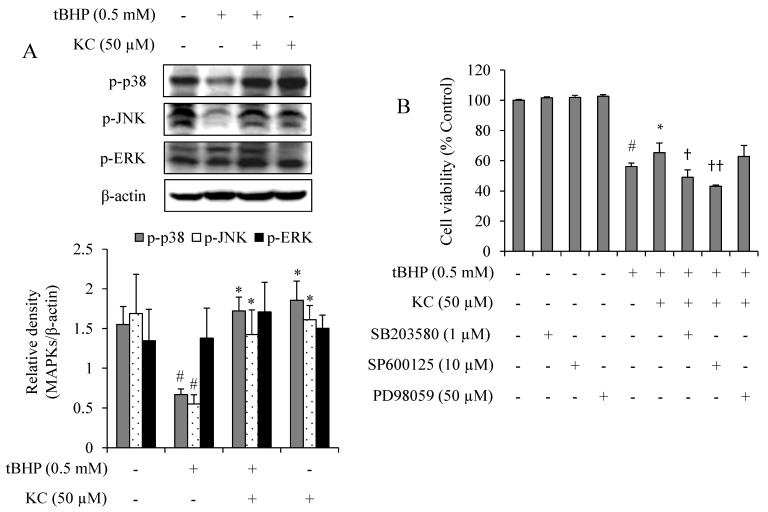
Effect of kushenol C on MAPKs expressions in tBHP-treated HEPG2 cells. (**A**) Cells were preincubated with or without KC for 1 h and then treated with or without 0.5 mM of tBHP for 12 h. p38, JNK, and ERK with their activated forms were investigated by Western blot assay and the band densities were analyzed using ImageJ analysis software, with respect to actin. (**B**) Cells were preincubated with KC at different concentrations and/or p38, JNK, and ERK inhibitors for 1 h and then treated with tBHP for 12 h. Cell viability was determined using EZ-Cytox reagent. Error bars represent the mean ± SD, # *p* < 0.001 vs. Control, * *p* < 0.05 vs. tBHP-only treated cells, † *p* < 0.01, †† *p* < 0.001 vs. tBHP plus KC treated cells.

The effects of KC on the activation of p38, ERK and JNK in tBHP-treated cells were investigated. As reported in Figure 5, there was a decrease in the expression of activated p38 (p-p38) and JNK (p-JNK), except for ERK (p-ERK), in HEPG2 cells treated with tBHP alone. However, when the cells were preincubated with 50 µM of KC before treatment with tBHP, a significant increase in the expressions of p-p38 and p-JNK, except for p-ERK, could be observed. In addition, treatment of the cells with KC alone maintained the expression levels of p-p38, p-JNK, and p-ERK when compared to the control cells. Further investigations with potent inhibitors of p38 (SB203580), JNK (SP600125), except for ERK (PD98059), revealed that while KC prevented tBHP-induced cell death, copreincubation of the tBHP-treated cells with KC and SB203580, SP600125, except for PD98059, at noncytotoxic concentration did not prevent tBHP-induced cell death.

### 3.7. KC Protected against APAP-Induced Liver Injury

**Figure 6:**
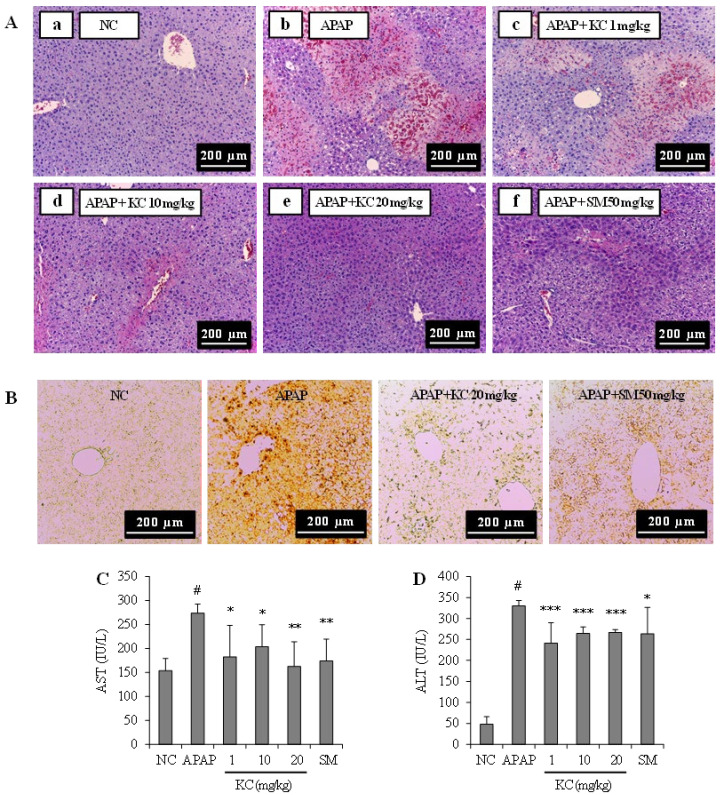
Effect of kushenol C on APAP-induced hepatotoxicity in mice. Thirty mice were partitioned into six groups (n = 5): group 1, normal control; group 2, APAP 500 mg/kg; group 3, APAP plus KC 1 mg/kg; group 4, APAP plus KC 10 mg/kg; group 5, APAP plus KC 20 mg/kg; group 6 (a positive control), APAP plus silymarin (SM) 50 mg/kg. (**A**) Hematoxylin and eosin-stained sections of the mice liver showing hepatotoxicity and inflammation. (**B**) Immunohistochemically stained sections of the liver showing the expression of cleaved caspase 3. (**C**,**D**) AST and ALT levels were measured in the serum of each mouse. Error bars represent the mean ± SD, # *p* < 0.001 vs. NC group, * *p* < 0.05, ** *p* < 0.01, *** *p* < 0.001 vs. APAP-only treated group.

The effects of KC on APAP-induced hepatotoxicity were investigated. The mice were coadministrated with KC and APAP. Histological results of liver sections presented in Figure 6A revealed massive necrosis and inflammation in the mice treated with APAP alone. KC treatment significantly ameliorated APAP-induced liver injury in a dose-dependent manner. Further findings through immunohistochemistry analysis demonstrated that cleaved caspase-3 was highly expressed in the mice treated with APAP alone while cotreatment with KC significantly decreased cleaved caspase-3 expression (Figure 6B). The serum levels of both AST and ALT, the markers of liver damage, were also markedly elevated in mice treated with APAP, and KC cotreatment significantly inhibited APAP-induced AST and ALT levels in the serum (Figure 6C,D). The results obtained with KC treatment were similar to the results obtain with cotreatment with silymarin, a positive standard used in the analysis.

### 3.8. KC Treatment Reduced APAP-Induced Hepatic Oxidative Stress

**Figure 7:**
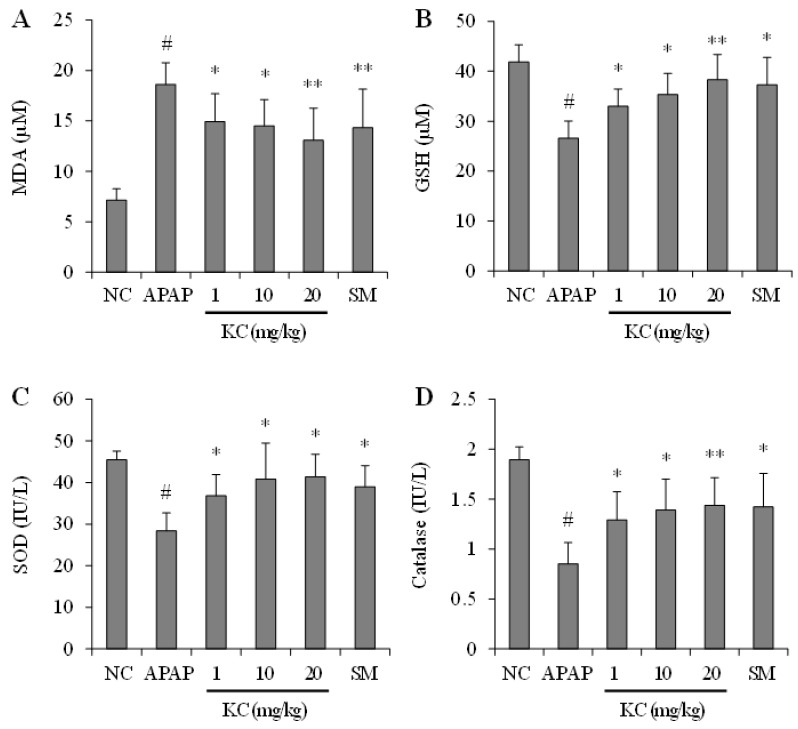
Effect of kushenol C on APAP-induced MDA production and suppression of antioxidant enzymes in mice. Thirty mice were partitioned into 6 groups (n = 5): group 1, normal control; group 2, APAP 500 mg/kg; group 3, APAP plus KC 1 mg/kg; group 4, APAP plus KC 10 mg/kg; group 5, APAP plus KC 20 mg/kg; group 6 (a positive control), APAP plus silymarin (SM) 50 mg/kg. (**A**) MDA levels, GSH (**B**), SOD (**C**), and catalase (**D**) were measured in the liver tissue. Error bars represent the mean ± SD, # *p* < 0.001 vs. NC group, * *p* < 0.05, ** *p* < 0.01 vs. APAP-only treated group.

The effects of KC on APAP-induced-induced oxidative stress in the liver were investigated. Antioxidant enzymes in liver tissues were investigated. As shown in Figure 7, hepatic MDA contents were increased in mice administered with APAP alone. The hepatic MDA was significantly decreased in the mice that were coadministered with KC at various concentrations and APAP. Silymarin, as expected, also decreased MDA levels to a level similar to KC administration. In addition, mice administered with APAP alone had decreases in the levels of GSH, SOD and catalase. Hepatic GSH, SOD and catalase were significantly increased in the mice that were coadministered with KC at various concentrations and APAP. Silymarin, as expected, also increased GSH, SOD and catalase levels to a similar level as KC.

### 3.9. KC Inhibited APAP-Induced Hepatic Inflammation

**Figure 8:**
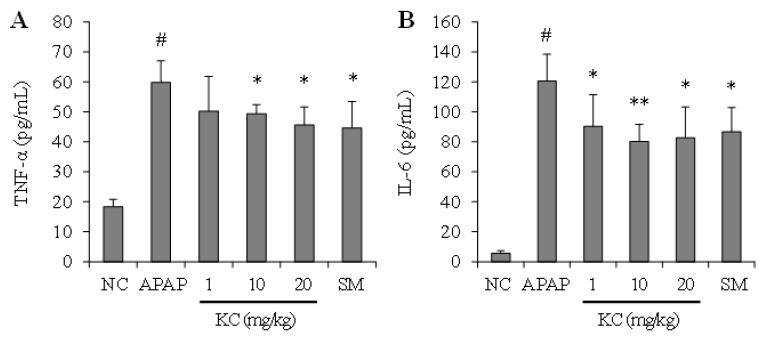
Effect of kushenol C on APAP-induced hepatic inflammation in mice. Thirty mice were partitioned into 6 groups (n = 5): group 1, normal control; group 2, APAP 500 mg/kg; group 3, APAP plus KC 1 mg/kg; group 4, APAP plus KC 10 mg/kg; group 5, APAP plus KC 20 mg/kg; group 6 (a positive control), APAP plus silymarin (SM) 50 mg/kg. TNF-α (**A**) and IL-6 (**B**) levels were measured in the serum of each mice. Error bars represent the mean ± SD, # *p* < 0.001 vs. NC group, * *p* < 0.05, ** *p* < 0.01 vs. APAP-only treated group.

The effects of KC on APAP-induced hepatic inflammation were investigated. As shown in Figure 8, TNF-α and IL-6 were significantly increased in the serum of mice administered with APAP alone. Cotreatment with KC significantly decreased the APAP-induced increase in TNF-α and IL-6 in the serum. The results obtain with KC treatment were similar with the results obtain with silymarin treatment.

## 4. Discussion

In the present study, we investigated the effects of KC in tBHP-treated HEPG2 cells and APAP-administered mice. We found that treatment of HEPG2 cells with tBHP induced the production of ROS that probably resulted in apoptosis and death of the HEPG2 cells due to oxidative stress. We also found that administration of APAP to mice led to oxidative stress, inflammation and hepatotoxicity of the liver. Preincubation of the HEPG2 cells with KC prevented the cell death caused by tBHP. Coadministration of the mice with KC and APAP also prevented oxidative stress, inflammation and hepatotoxicity caused by APAP.

tert-Butyl hydroperoxide (t-BHP) is commonly used as a model substance to study cellular alterations and outlining mechanisms resulting from oxidative stress in cells. It is specifically used as an in vitro model to study nonalcoholic fatty liver disease and hepatotoxicity because it induces ROS production and depletes GSH, thus resulting in oxidative stress and ultimately death of liver cells [11]. Here, we revealed that KC at noncytotoxic concentrations markedly enhanced the HEPG2 cell viability and prevented apoptosis by probably suppressing ROS production. ROS induces the release of apoptogenic factors like caspases from mitochondria. These caspases are a family of cysteine proteases that cleave target proteins at specific residues. Among the more than ten members of the caspase family, the extensively studied caspase-3 (the “executor of apoptosis,”) plays a crucial role in cell death [12,13]. The caspase protein is usually preserved in the cell in an inactive form (pro-caspase). Upon activation it is cleaved and converted to its active form (cleaved caspase) that executes the apoptosis of cells. In this study, we demonstrated that tBHP treatment of HEPG2 cells led to the decrease in pro-caspase 3, probably suggesting an increase in its cleaved form (the cleaved caspase 3). KC treatment of the tBHP-treated HEPG2 cells helped to maintain the level of pro-caspase 3 as in the control cells, thus suggesting that there was a decrease in the level of the cleaved caspase 3 in the cells. We predict that this regulation mechanism of KC and procaspase expression helped in the prevention of apoptosis and cell death of tBHP-treated HEPG2 cells. However, this fact will need to be confirmed in further studies by measuring cleaved caspase 3. ROS production in cells also affects the expression of other apoptosis related proteins such as the antiapoptosis protein-Bcl-2 and proapoptosis protein-Bax that can also cause DNA damage and cell death [14]. Here, we revealed that prevention of tBHP-induced apoptosis by KC was also evidenced by the regulation of mitochondrial-related apoptosis signals, which included upregulation of the antiapoptosis protein Bcl-2 expression, the suppression of expression of the proapoptosis protein-Bax, and prevention of DNA damage revealed by increase in the expression of OGG1. Here, we determined that KC acts by favoring the relative abundance of antiapoptotic proteins over their proapoptotic partners. We also specifically determined that tBHP-induced cell death was associated with DNA damage because potent inhibitors of OGG1 failed to prevent cell death. The fact that cotreatment with KC and OGG1 inhibitor prevented cell death meant that one of the mechanisms of action of KC was preventing DNA damage in the HEPG2 cells. KC was also found to upregulate the expression GSH in the tBHP-treated cells, which might have also contributed to the prevention of the oxidative stress and HEPG2 cells death induced by tBHP. One could conclude here that KC’s protection of tBHP-induced cell death is mediated by an increase in cellular GSH biosynthesis.

In response to oxidative stress, the antioxidant defense system is activated as a compensatory response to protect cell damage by maintaining cellular redox homeostasis [15]. The activation of the Nrf2 transcription factor through the AKT signaling pathway is responsible for the cellular biosynthesis of antioxidant enzymes in the cells. Here, we further determined whether KC’s upregulation of GSH and OGG1 was mediated by its regulation of the AKT/Nrf2 pathway. We found that tBHP suppressed the activation of AKT (p-AKT) and Nrf2 expression, indicating a decrease in activated Nrf2 in the cells. However, KC enhanced the activation of AKT and Nrf2 expression, thus indicating that KC’s action in the AKT/Nrf2 pathway promoted the expression of Nrf2 downstream antioxidants (GSH and OGG1) and hence prevention of cell death caused by tBHP. This conclusion was further confirmed because potent inhibitors of the AKT/Nrf2 pathway failed to prevent cell death in the presence of KC. However, it is thought that further investigation into the mechanism of KC on the nrf2 signaling pathway is needed.

To further under the mechanisms underlying the protective effects of KC in tBHP-treated HEPG2 cells, we investigated the effect of KC on the activation of ERK, JNK and p38 in the MAPK pathway. Oxidative stress can activate various cellular kinases, including MAPKs, which are responsible for cell protection against oxidative stress and inflammation [16,17]. Here, we found that tBHP treatment instead led to the suppression of the activation of JNK and p38, contrary to other studies [18,19]. We concluded that this decrease in the levels of activated JNK and p38, except for ERK, was the results of cell death induced by tBHP. Our conclusion was confirmed when KC treatment of the tBHP cells prevented cell death but KC treatment of the tBHP cells in the presence of potent inhibitors of JNK and p38 did not rescued the cell death caused by tBHP. The data further suggest that the activation of MAPKs might be involved in the cytoprotective effect of KC in tBHP-treated cells.

The protective effects of KC in HEPG2 cells pushed us to further investigate in vivo, the effects of KC on APAP-induced hepatotoxicity in mice. APAP overdose is well known for its toxicity to liver where it even causes liver failure in experimental animals and humans [20]. Numerous studies have demonstrated that oxidative stress plays a key role in APAP-induced hepatotoxicity [20,21,22]. In our in vivo studies we found that APAP also induced hepatotoxicity and damage to the liver as evidenced by intrahepatic hemorrhage; increased cleaved caspase 3 expression; liver inflammation, liver cell vacuolar degeneration, increase levels of AST, ALT and MDA, and decreases in GSH, SOD and catalase in the liver. We also observed that KC had therapeutic potential in detoxification against APAP-induced intoxification and hepatotoxicity of the liver. This was evidenced by reduced or absence of intrahepatic hemorrhage, decreased expression of cleaved caspase 3; and reduced levels of AST, ALT and MDA in mice co-administered with KC. The upregulation of the GSH, SOD and catalase antioxidant defense system further confirmed the protective potentials of KC against oxidative stress that leads to hepatotoxicity in the liver. In addition, the decreased level of proinflammatory cytokines such as IL-6 and TNF-α might also contribute to the amelioration of APAP-induced hepatic inflammation. These data indicated that KC protected against APAP-induced liver injury and the effects of KC, even at lower doses, was almost the same as silymarin, the well-known antidote used for treatment of liver injury [23,24], thus revealing the potential need for further investigation of KC as antidote for APAP-induced acute liver injury.

In conclusion, the findings of this study demonstrated that pretreatment of HEPG2 cells with KC protects against tBHP-induced oxidative stress by apoptosis and cell death through the regulation of caspase 3, Bax/Bcl-2, GSH and ROS generation, through a mechanism that involves Nrf2 activation mediated by AKT. The study also demonstrated the therapeutic detoxification of KC against APAP-induced hepatotoxicity, revealing the great potential of KC for the treatment of liver injury caused by APAP overdose.

## Data Availability

The data used to support the findings of this study is included within the article, and the data are available from the corresponding author upon request.
